# Triple DMARD treatment in early rheumatoid arthritis modulates synovial T cell activation and plasmablast/plasma cell differentiation pathways

**DOI:** 10.1371/journal.pone.0183928

**Published:** 2017-09-01

**Authors:** Alice M. Walsh, Mihir D. Wechalekar, Yanxia Guo, Xuefeng Yin, Helen Weedon, Susanna M. Proudman, Malcolm D. Smith, Sunil Nagpal

**Affiliations:** 1 Immunology, Janssen Research and Development, LLC., Spring House, Pennsylvania, United States of America; 2 Repatriation General Hospital, Daw Park, South Australia, Australia; 3 Flinders University, Adelaide, South Australia, Australia; 4 Rheumatology Unit, Royal Adelaide Hospital, Adelaide, South Australia, Australia; 5 Discipline of Medicine, University of Adelaide, Adelaide, South Australia, Australia; Keio University, JAPAN

## Abstract

**Objectives:**

This study sought to investigate the genome-wide transcriptional effects of a combination of disease modifying anti-rheumatic drugs (tDMARD; methotrexate, sulfasalazine and hydroxychloroquine) in synovial tissues obtained from early rheumatoid arthritis (RA) patients. While combination DMARD strategies have been investigated for clinical efficacy, very little data exists on the potential molecular mechanism of action. We hypothesized that tDMARD would impact multiple biological pathways, but the specific pathways were unknown.

**Methods:**

Paired synovial biopsy samples from early RA patients before and after 6 months of tDMARD therapy were collected by arthroscopy (n = 19). These biopsies as well as those from subjects with normal synovium (n = 28) were profiled by total RNA sequencing.

**Results:**

Large differences in gene expression between RA and control biopsies (over 5000 genes) were identified. Despite clinical efficacy, the expression of a restricted set of less than 300 genes was reversed after 6 months of treatment. Many genes remained elevated, even in patients who achieved low disease activity. Interestingly, tDMARD downregulated genes included those involved in T cell activation and signaling and plasmablast/plasma cell differentiation and function.

**Conclusions:**

We have identified transcriptomic signatures that characterize synovial tissue from RA patients with early disease. Analysis after 6 months of tDMARD treatment highlight consistent alterations in expression of genes related to T cell activation and plasmablast/plasma cell differentiation. These results provide novel insight into the biology of early RA and the mechanism of tDMARD action and may help identify novel drug targets to improve rates of treatment-induced disease remission.

## Introduction

Rheumatoid arthritis (RA) is a common autoimmune disease characterized by synovitis, systemic inflammation, and the presence of autoantibodies. RA causes progressive joint damage, disability, and significant reduction in health-related quality of life [1]. Treatment guidelines for RA typically recommend initial treatment with conventional synthetic disease-modifying anti-rheumatic drugs (DMARDs), primarily methotrexate (MTX), which results in low disease activity in approximately 30% of patients [2–4]. For patients whose disease is not well-controlled by MTX, anti-tumor necrosis factor (TNF) biologic DMARDs are commonly prescribed [5, 6]. Alternatively, a combination of triple conventional synthetic DMARDs (tDMARD; MTX, sulfasalazine and hydroxychloroquine) may be used, which has shown remarkable efficacy in early RA patients [4]. Studies in patients with active early RA or established RA after MTX failure have demonstrated that the efficacy of tDMARD therapy may approach the levels achieved by anti-TNF agents [2, 4, 7]. However, anti-TNF agents may have an edge over combination DMARDs in terms of their rapid mode of action. Infliximab showed better reduction in disease score and radiographic progression compared to tDMARD therapy initially, but the difference was not maintained after two years of treatment [7]. These results bode well for treatment decisions in the developing world, where cost considerations preclude the use of biologics on many suffering patients.

Unfortunately, many RA patients do not achieve low disease activity or remission with current therapies (both conventional synthetic DMARDs and biologic DMARDs) and may cycle through multiple treatment regimes. Although more treatment options are becoming available to patients (e.g., small molecule Janus kinase (JAK) inhibitors, interleukin-6-inhibitors), there is insufficient mechanistic understanding differentiating these therapies. Without this differentiation, it will be difficult to rationally inform treatment decisions and avoid unnecessary trial-and-error when treating patients. New molecular datasets from disease-relevant synovial tissue have the potential to improve the understanding of these therapies. Additionally, data from RA patients treated with different therapies could help identify novel drug targets for patients that are resistant to therapy. These data could also be used to identify rational combinations with currently available drugs.

Despite several studies demonstrating sustained efficacy of tDMARD in RA [2, 4, 7, 8], the molecular basis of its therapeutic action is lacking. Increased understanding of tDMARD action in treatment-naïve early RA may identify altered pathways in the synovium, which could provide clues to the identity of novel drug targets for prevention and treatment of disease. In this study, we investigated the global molecular effects of tDMARD therapy in synovial tissue biopsy samples obtained prospectively (at baseline and 6 months post-treatment) from treatment-naïve early RA patients by transcriptional profiling. Comparison of RA biopsies before and after treatment and the comparison of the baseline early RA synovial tissue with those obtained from normal healthy subjects were performed. Our analysis identified large differences in gene expression between early RA and healthy synovial tissue biopsies. After 6 months of tDMARD treatment, the expression of a restricted set of genes related to the immune system and largely expressed by lymphocytes were reversed. However, most genes remained elevated, even in patients that achieved good clinical response to therapy. Finally, this study revealed that tDMARD treatment resulted in inhibition of the expression of T cell activation and plasmablast/plasma cell differentiation genes, indicating that a small subset of genes in these two pathways may explain the molecular underpinnings of tDMARD therapeutic action in early RA.

## Materials and methods

### Human subjects

Synovial biopsy specimens for healthy donors were obtained from patients with knee pain attending a sports medicine day surgical facility. Healthy subjects were defined as those who had no evidence of any form of arthritis on history or examination and had no cartilage damage or synovitis on knee arthroscopy.

Paired longitudinal arthroscopic synovial biopsies were collected from patients with early RA (defined as within 12 months of onset of symptoms) at baseline and after 6 months of treatment with tDMARDs administered per a treat-to-target protocol. Patients were sero-positive, DMARD naïve, and fulfilling the 1987 revised American College of Rheumatology (ACR) and/or the 2010 ACR/ European League Against Rheumatism (EULAR) criteria. Demographic and patient characteristics are given in Table 1.

**Table 1:** Patient information.

	Early RA baseline	Early RA 6 months	Healthy/Normal
Number of subjects	19	19	28
Sex—Female (%)	8 (42%)		10 (36%)
Age (Years)	Mean: 50.84		Mean: 35.18
	Min: 33		Min: 13
	Max: 72		Max: 73
Disease duration at baseline (weeks)	Mean: 17.58		n/a
	Min: 4		
	Max: 40		
CCP (U/ml)	Min: 11 (11/19 with value >100)		n/a
RF (IU/ml)	Mean: 130		n/a
	Min: 16 (2/19 RF-negative)		
	Max: 570		
CRP (mg/dl)	Mean: 2.41	Mean: 0.60	n/a
	Min: 0.069	Min: 0.034	
	Max: 9.2	Max: 4.6	
ESR (mm/hr)	Mean: 39.42	Mean: 14.42	n/a
	Min: 7	Min: 1	
	Max: 108	Max: 52	
DAS-28 (ESR)	Mean: 5.80	Mean: 3.10	n/a
	Min: 4.80	Min: 1.08	
	Max: 7.40	Max: 5.56	
HAQ-DI	Mean: 1.20	Mean: 0.53	n/a
	Min: 0	Min: 0	
	Max: 2	Max: 1.375	

n/a: not applicable

All protocols for collecting synovial biopsies and blood were approved by the local Institutional Review Board, Flinders Medical Centre and Flinders University Ethics Committee. All patients gave written informed consent for participating in the study.

### RNA-sequencing

Total RNA was extracted from RA synovial samples and the quality of all RNA samples was evaluated using an Agilent Bioanalyzer. Sequencing libraries were prepared using TruSeq Stranded Total RNA RiboZero protocol from Illumina. Libraries were pooled and sequenced with an Illumina HiSeq 2000 with paired-end 100 bp flow cells. Raw read quality was evaluated using FastQC.

Reads were trimmed for adaptors and sequence quality. The average number of clusters (post-trimming) per sample was > 80 million. Trimmed reads were aligned to human b37.3 reference genome using the STAR v2.4 aligner [9]. Aligned reads were quantified using RSEM v1.2.14 algorithm [10] with UCSC transcriptome model (accessed on 03/17/2014) that included lincRNAs from Ensembl v75. This transcriptome model has a total of 34495 genes and 88933 isoforms. Aligned data was evaluated for quality using several quality metrics (e.g., mapping rate, coverage) and visually inspected for deviation from the population across multiple metrics and principal components analysis.

Data are available publicly through NCBI GEO database (Healthy samples are under accession number GSE89408. RA samples are under accession number GSE97165).

### Statistics

Statistical analysis of RNA-seq data was performed in R with the “limma” package [11]. Counts were converted to log2 counts per million (cpm), quantile normalized and precision weighted. Gene features were filtered to those with cpm > 1 in at least one sample (17,768 genes). A linear model was fit to each gene, and empirical Bayes moderated t-statistics were used to assess differences in expression.

RNA-seq features were considered differentially expressed if they satisfied a 2 fold-change and 0.05 adjusted p-value cutoff (FDR) unless otherwise specified. The Benjamini-Hochberg method was used to calculate p-values adjusted for multiple hypotheses.

### Pathway enrichment analysis

Gene set enrichment was calculated with a publicly available database of pathway terms (www.reactome.org) using a hypergeometric test. P-values were adjusted for multiple hypotheses using Bonferroni correction.

Upstream regulators were inferred using the Ingenuity Pathway Analysis (IPA) tool and knowledgebase. To reduce the number of input features, only features with log2(fold-change) > 1.5 up or down were included. Detailed descriptions of IPA analysis are available on the IPA website (http://www.ingenuity.com).

### Co-expression module analysis

Co-expression modules were derived using the weighted gene co-expression network analysis (WGCNA) algorithm in R with the “WGCNA” package [12]. All samples and all gene features used in the differential expression analysis were also used to generate co-expression modules. A soft-thresholding power of 6 was used based on testing a range of powers and selecting a power to optimize scale-free topology and mean connectivity. The default parameters of the *blockwiseModules* function for minimum module size and tree cutting were used. Modules were summarized by their eigengenes (first principal component). If the variance explained by the eigengene was less than 40%, that module eigengene was not included in downstream analysis (module 1 in this analysis).

## Results

### Transcriptomics of synovial biopsies captures large differences between normal and diseased joints

Synovial biopsies were obtained by knee arthroscopy from 28 healthy individuals and 19 recently diagnosed early RA patients at baseline and after 6 months of treatment with a triple DMARD (tDMARD) regimen. All RA patients were treatment naive at the time of baseline synovial biopsy and had disease duration of less than 12 months, from onset of symptoms.

**Fig 1:**
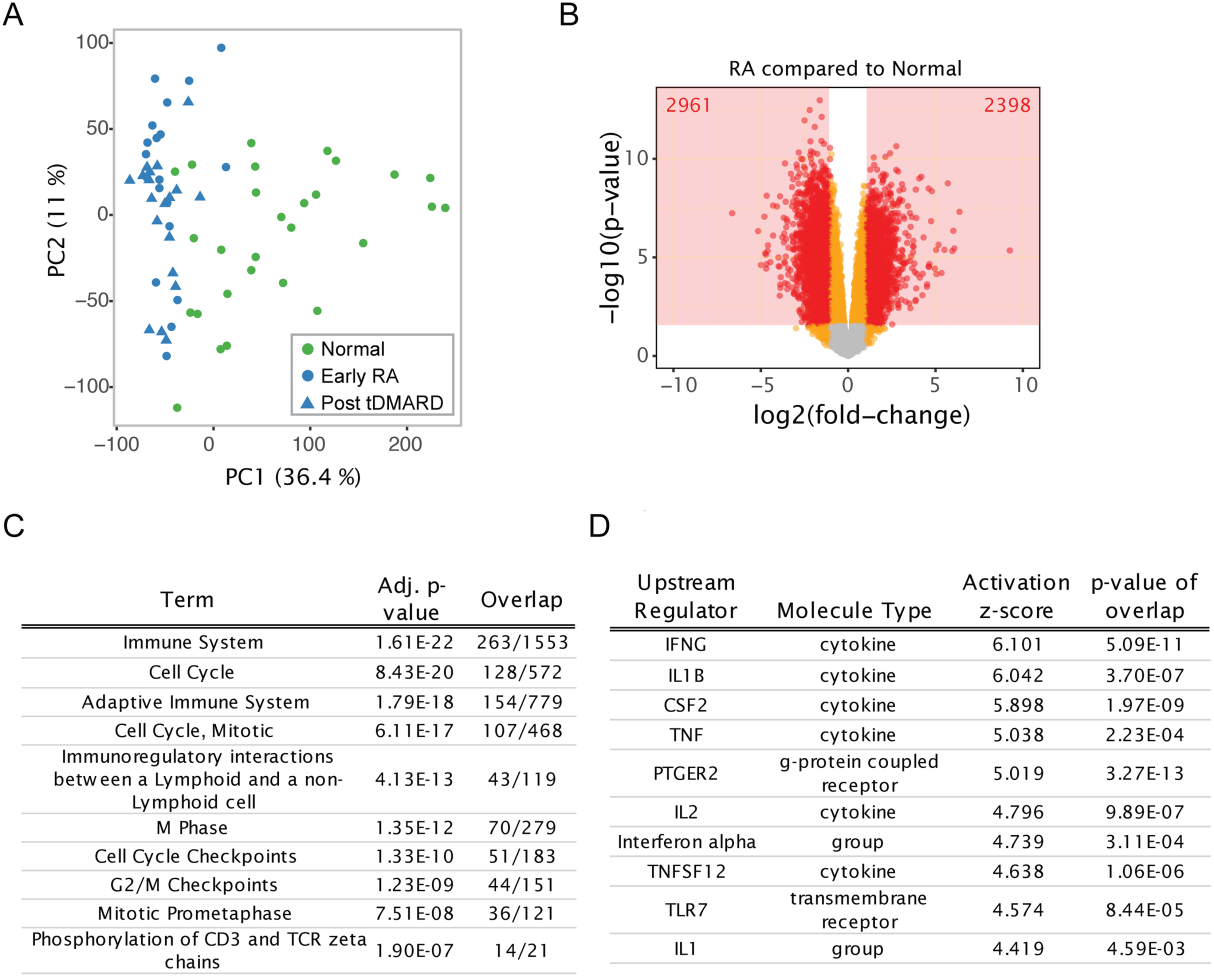
Large transcriptional differences separate RA synovial biopsies from healthy samples. (A) PCA demonstrates variability of normal synovial biopsy samples and clustering of RA samples together. Sample scores are plotted for the first two principal components (the percent variance explained is given for each PC). Samples are colored by disease type; shape indicates visit for RA subjects. PCA was performed with all gene expression features used in this analysis. (B) Volcano plot depicting the log(fold-changes) and p-values for the comparison of all baseline RA samples to all normal samples. The number of gene features satisfying the statistical significance cutoff are given. (C) Top ten pathway terms (Reactome database) and (D) top ten predicted upstream regulators (IPA database) are shown from the comparison of baseline RA samples to normal samples.

Biopsy samples were profiled for transcriptome-wide differences by total RNA sequencing. RA samples clustered separately from the healthy samples by principal component analysis (PCA; Fig 1A). Comparisons between groups (adjusting for sex) identified many differentially expressed genes. 2398 and 2961 genes were higher and lower, respectively, in RA versus healthy subjects (Fig 1B). Gene features with false discovery rate (FDR) < 5% and magnitude of fold-change > 2 were considered differentially expressed. Before undertaking deeper analysis of the transcriptional changes, we examined trends in the genes differentially expressed in baseline samples. Enrichment for canonical pathways and potential upstream regulators based on literature databases is shown in Fig 1C and 1D. The top enriched pathways were distributed between those involved in cell cycle/proliferation and the immune system (Fig 1C). The top predicted activated upstream regulators were largely cytokines involved in immune response (e.g., IFNG, IL1B, TNF) (Fig 1D).

### The expression of a restricted subset of transcriptional markers is modified by tDMARD treatment

**Fig 2:**
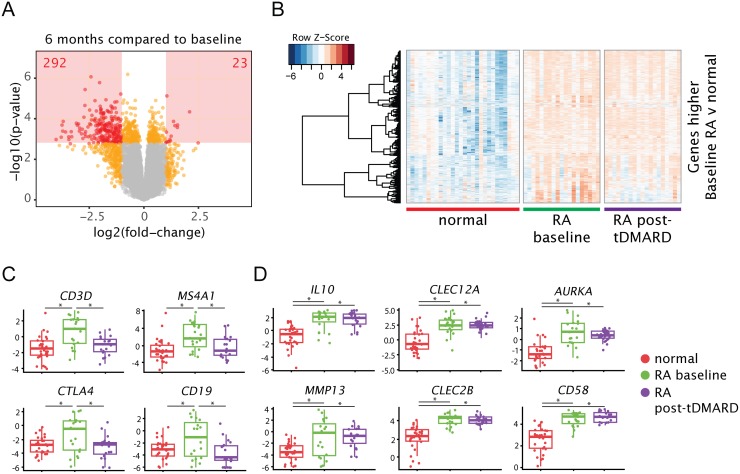
Genes selectively expressed by lymphocytes are decreased after 6 months of tDMARD treatment, while other genes remain elevated compared to normal joints. (A) Volcano plot depicting the log(fold-changes) and p-values for the comparison of the post-treatment RA samples to the corresponding baseline samples. The number of gene features satisfying the statistical significance cutoff are given. (B) Heatmap of genes that met significance criteria for differential expression between baseline RA samples and normal samples. Rows are variance scaled. Samples are ordered along the x-axis, while genes are clustered on the y-axis. (C) Representative genes expressed by T and B lymphocytes. (D) Representative genes that were elevated in baseline RA samples, but not decreased post-tDMARD treatment. Plotted values are quantile-normalized log2-cpm. For each group, all samples are plotted in addition to box-plots summarizing the group. * indicates adj. p < 0.05.

We next evaluated transcriptional changes in 19 early RA patients after 6 months of tDMARD treatment. Eleven patients achieved “good” and six patients achieved “moderate” response as defined by EULAR response criteria based on DAS28-ESR (disease activity score using 28 joints and erythrocyte sedimentation rate) scores. Despite clinical response, biopsy transcriptional profiles did not return to a “normal-like” state post-treatment. Differences were tested within subject between pre- and post-treatment samples. 23 genes were significantly increased while 292 genes were significantly decreased by tDMARD treatment (Fig 2A). Most of the genes elevated relative to normal samples at baseline remained elevated post-treatment, while a focused set of genes decreased (146 decreased out of 2398 genes; Fig 2B). Several of the genes decreased following tDMARD treatment are well-known lymphocyte markers (Fig 2C) such as *CD3D* and *CTLA4* on T cells and *MS4A1* (CD20) and *CD19* on B cells. Several representative genes elevated at baseline in RA samples but not decreased by tDMARD treatment are shown in Fig 2D. These included genes expressed by innate immune cells and antigen presenting cells (*CLEC2B*, *CLEC12A*, *CD58*), cell cycle genes (*AURKA*), and other genes with relevance to RA pathophysiology (*IL10*, *MMP13*).

### Early RA synovial biopsies can be grouped into three subtypes, but these subtypes are not associated with clinical features

**Fig 3:**
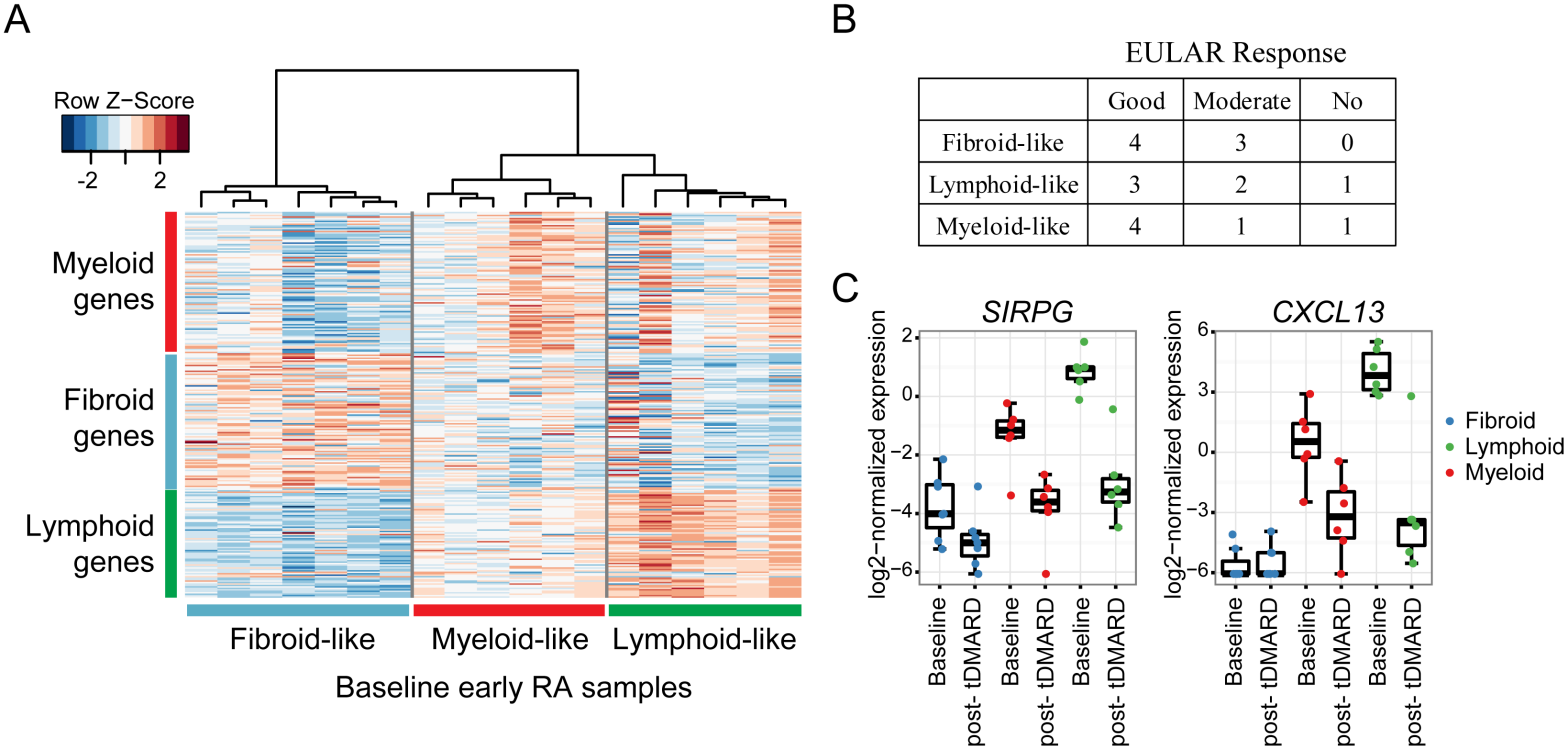
RA samples can be grouped into subtypes based on previously identified gene expression features. (A) Hierarchical clustering of baseline early RA samples using features previously published (Dennis et al., 2014 [13]). (B) Table of EULAR response after 6 months of tDMARD treatment based on inferred synovial subtypes at baseline. (C) Expression of example genes grouped by visit and synovial subtype.

Consistent with the known heterogeneity of RA synovial tissue [13, 14], some RA samples did not have elevated expression of T cell and B cell markers at baseline (Fig 2C), suggesting that these biopsies lacked a high level of infiltration by T and B cells. Previous studies have suggested that levels of immune cell infiltration can categorize synovial biopsies into subtypes (referred to here as fibroid, myeloid, lymphoid), which may be associated with response to therapeutics [13]. In this study, the baseline early RA samples could be categorized into subtypes using previously published genes and hierarchical clustering (Fig 3A). However, the inferred tissue subtypes were not associated with clinical response to tDMARDs (Fig 3B). The inferred subtypes were also not associated with disease activity or any other factors available at baseline (HAQ-DI, DAS28-ESR, age, sex, disease duration). We tested whether any of the pharmacodynamic changes (post-treatment versus pre-treatment) observed differed among synovial subtypes. *SIRPG* is an example of a gene whose expression decreased independent of subtype (Fig 3C). Several genes that were not highly expressed in fibroid-like biopsies (e.g., *CXCL13*), were therefore only modulated in myeloid- and lymphoid-like biopsies (Fig 3C). This analysis suggests that synovial subtypes based on immune infiltration are important factors in analysis of synovial biopsies, but that their relevance to currently accepted clinical measurements have not been established.

### Co-expression modules annotate differences in gene expression across sample properties

Because gene expression is highly coordinated and cell-context specific, evaluation of gene transcriptional changes using co-expression methods can identify functionally-related modules of genes. We used the WGCNA algorithm [12] to derive 20 modules of co-expressed genes in our dataset. We hypothesize that these modules represent genes with related function and common transcriptional regulation, or genes that are selectively expressed in certain cell types. Instead of examining the pattern of expression of thousands of individual genes, we instead considered the pattern of these 20 modules. The modules were annotated manually with potential function and cell type using several databases (complete lists of genes in each module are provided in S1 Table).

**Fig 4:**
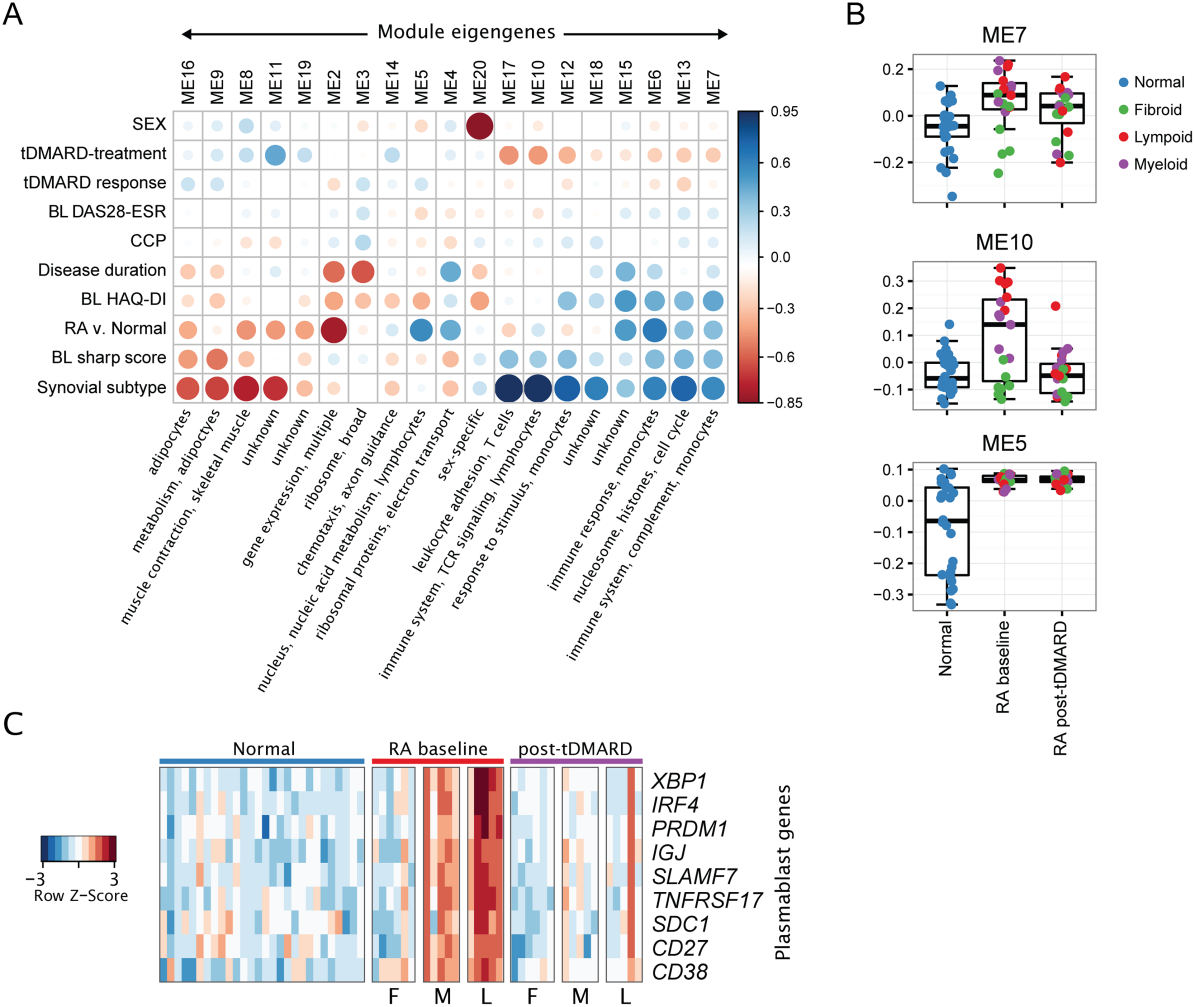
T cell activation and plasmablast/plasma cell differentiation pathways are down-modulated by tDMARD treatment in early RA synovial biopsies. (A) Co-expression modules are associated with disease and tDMARD treatment but not baseline DAS28-ESR or clinical response to tDMARDs. The spearman correlation coefficient between sample characteristics (y-axis) and module eigengenes (x-axis) are visualized. The size and color of each marker represents the correlation coefficient. Brief summarizations of the composition of each module are given. “BL” indicates baseline data was used. (B) Example module eigengenes are shown summarized by sample group. (C) Heatmap representation of plasmablast/plasma cell genes that are elevated in baseline RA samples and reduced by tDMARD treatment. The expression of these genes in normal samples is also shown. Samples are sorted on the x-axis by disease type, treatment, and inferred subtype (F: fibroid, M: myeloid, L: lymphoid).

We tested for association between sample characteristics and the 20 co-expression modules summarized by module eigengenes (Fig 4A). No modules were highly associated in baseline RA samples with DAS28-ESR, clinical response to tDMARD treatment, or anti-citrullinated peptide antibody (ACPA) titer. However, multiple modules were associated with disease state (RA versus normal) and other disease measures (baseline health assessment questionnaire disability index (HAQ-DI) and radiographic damage (van der Heijde modified Sharp score)). The modules elevated in RA compared to non-RA samples (e.g., modules 5 and 6) were annotated with functions related to the immune system and contained genes highly expressed in immune cells. The modules decreased in RA (e.g., modules 2, 8, and 11) were annotated with non-immune functions and cell types such as muscle and adipocytes. However, likely because of the variability of RA samples, some modules were not highly associated with RA disease when considering all baseline RA samples together, but instead were highly associated with synovial subtype. For example, the expression of modules 10, 12, and 17 were variable in baseline RA samples with higher expression in lymphoid and myeloid subtypes than fibroid subtype samples.

There was a trend that modules increased in baseline RA compared to healthy samples also showed a decrease post-treatment (likewise baseline decreased modules showed an increase post-treatment). For example, module 7 is increased in baseline RA samples and then decreased by tDMARD treatment (Fig 4B). This module is enriched for genes related to immune system biology and genes highly expressed in monocytes and macrophages (includes *CD14*, *CD74*, *CXCL1*, HLA genes, *IL10RA*, *MMP1*, *TNF*). Module 10 is increased in baseline myeloid- and lymphoid-like RA samples and is decreased post tDMARD treatment (Fig 4B). Module 10 contains immune system genes that are enriched in T cell activation and differentiation pathways (e.g., *CD3D*, *ITK*, *CTLA4*, *LCK*, *RASGRP1*). On the other hand, module 5 is increased in RA samples, but not changed by tDMARD treatment (Fig 4B). Module 5 is enriched for genes involved in nucleic acid binding and genes related to transcription and gene expression.

### tDMARD treatment down-regulates the expression of plasmablast/plasma cell differentiation genes

We further examined the expression of genes involved in enriched canonical pathways “Immune System” and “Adaptive Immune System” (Fig 1) as well as co-expression module 10 (Fig 4A and 4B) and discovered that tDMARD treatment downregulated the expression of several genes involved in plasmablast/plasma cell differentiation, survival, and function. The expression of plasmablast surface markers, *CD38*, *CD27*, *CD138/SDC1*, and *SLAMF7* were upregulated in early RA synovial tissue biopsies compared to healthy synovium and down-regulated after tDMARD treatment (Fig 4C). As expected, the highest levels of these markers were observed in biopsies clustered into the lymphoid subtype, although many myeloid and some fibroid biopsies also exhibited elevated expression. X-box binding protein 1 (*XBP1*), interferon-regulatory factor 4 (*IRF4*) and PR domain zinc finger 1 (*PRDM1*) are master regulators of plasmablast differentiation. Similarly to that of *CD38* and *SLAMF7*, their expression was also upregulated in early RA synovium and downregulated after tDMARD treatment. Furthermore, the expression of *IGJ*, a marker of plasma cells, was increased in early RA and decreased in the synovial tissue post-tDMARD treatment (Fig 4C). Finally, the synovial expression of B-cell maturation antigen (*BCMA/TNFRSF17*), a critical survival receptor for plasma cells was also increased in early RA compared to normal controls and inhibited after treatment with tDMARD (Fig 4C). These results indicate that tDMARD may exert its therapeutic effect at least in part by inhibiting plasmablast/plasma cell differentiation and function. The single post-treatment biopsy sample with high levels of plasmablast genes came from a patient with high disease activity post-treatment (DAS28-ESR = 5.4 at 6 months) (Fig 4C).

### Transcriptional changes and annotated pathways that remain elevated in patients with low disease activity

**Fig 5:**
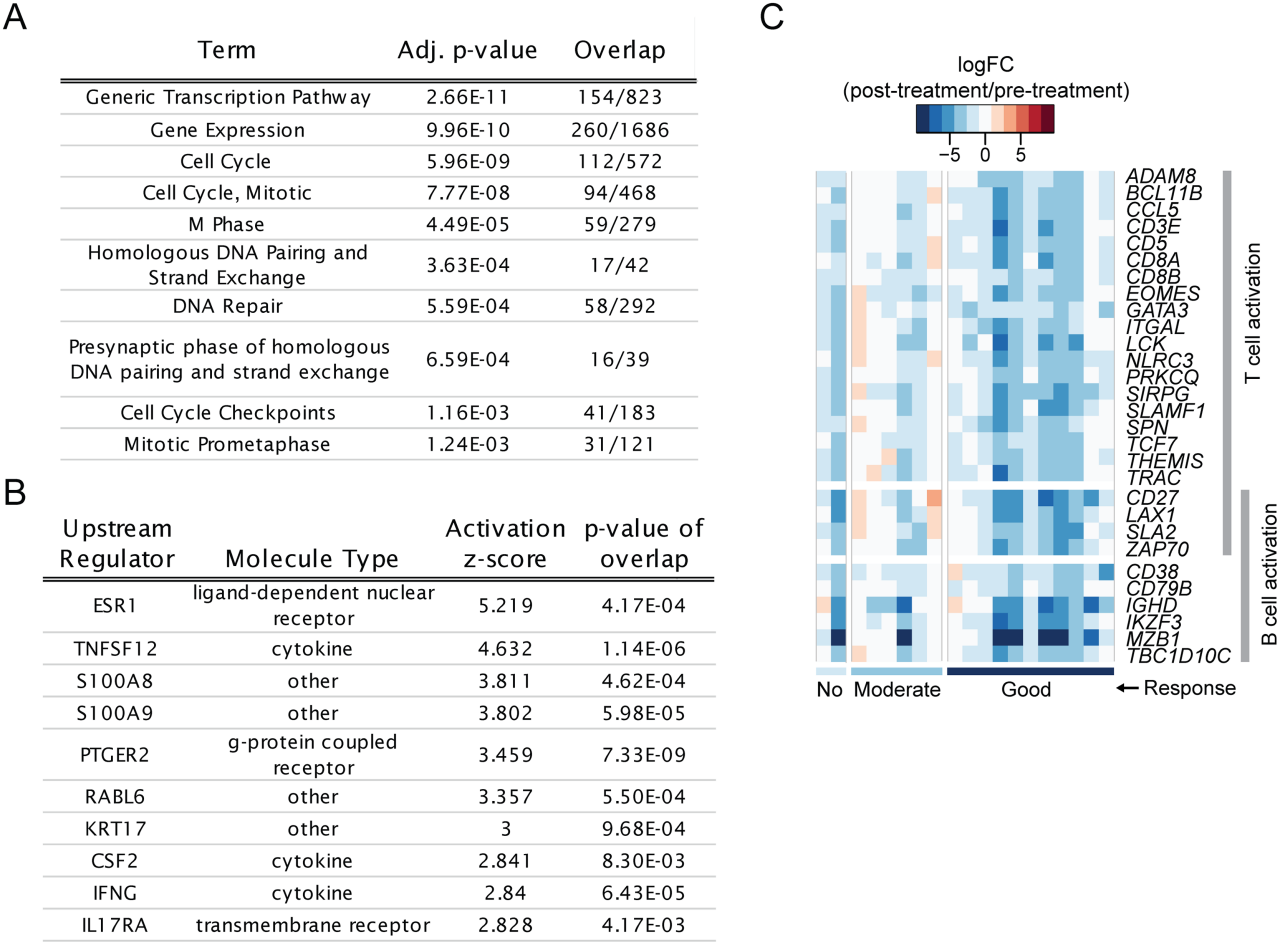
Several non-lymphocyte-related pathways remain elevated in samples from patients with LDA and lymphocyte activation genes tend to decrease more in patients with better response to tDMARDs. (A) Top ten pathway terms (Reactome database) and (B) top ten predicted activating upstream regulators (IPA database) are shown from the comparison of post-tDMARD LDA RA samples to normal samples. (C) Heatmap visualizing log(fold-changes) for each early RA subject post-tDMARD treatment compared to pre-treatment. Selected genes are those that were associated with treatment response and belonged to the “T cell activation” and/or “B cell activation” GO terms. Samples (x-axis) are ordered by EULAR response.

After 6 months of tDMARD treatment, 12/19 RA patients in this study met the criteria for low disease activity (LDA; defined as DAS28-ESR < 3.2). We compared these post-treatment, LDA samples to the healthy control samples to derive differentially expressed genes. These genes represent transcriptional differences that remain in RA joints even when a global disease activity measure indicates clinical disease suppression. The expression of 2781 and 3146 genes were increased and decreased, respectively, in LDA RA samples compared to healthy controls. Pathway enrichment with the Reactome database (www.reactome.org) indicated that the increased genes were over-represented in pathways involved in proliferation (gene expression, cell cycle, M phase) (Fig 5A; S2 Table). There was no significant enrichment for immune and inflammation pathways in agreement with the observed changes post-treatment described above. However, predicted activated upstream regulators (from Ingenuity Pathway Analysis) included pro-inflammatory cytokines/chemokines (e.g., TNFSF12 or TWEAK, IFNG, CSF2, S100A8, S100A9) (Fig 5B; S3 Table). The genes contributing to these predictions are selectively expressed in myeloid cells such as macrophages and monocytes (e.g., *S100A8*, *CCL19*, *CCR1*, *CCL4*, *TLR1*). Therefore, this result could reflect a remaining elevation of innate immune cell infiltration post-treatment.

### Clinical response to tDMARD is associated with greater down-modulation of T- cell activation genes

We tested whether the pharmacodynamic changes observed at 6 months were associated with clinical response to tDMARD treatment as defined by EULAR response criteria [15]. If genes/pathways whose expression were elevated in RA synovium were also decreased by tDMARD treatment and in a response-dependent manner, this would confirm the disease and treatment relevance of these genes/pathways. We treated EULAR response as a numerical value, where 0 was “no”, 1 was “moderate”, and 2 was “good” response. 106 genes had changes positively associated with response level, indicating that larger changes were seen in patients with greater responses. However, this approach was limited by a small sample size, particularly for non-responders (2 patients with “no response”). The genes with changes positively associated with response included many genes known to be involved in T-lymphocyte activation such as *PRKCQ* (PKCθ), *BCL11B*, *TCF7*, *EOMES*, *GATA3 ZAP70*, and *LCK* (Fig 5C). These results indicate that down-modulation of T cell activation pathway might be one of the mechanisms of tDMARD therapeutic action.

## Discussion

A number of studies have demonstrated the safety and efficacy of tDMARD treatment in early and established RA, including non-inferiority to anti-TNF agents [2, 4, 7, 8]. However, the molecular mechanisms of tDMARD action in RA synovial tissue have not been explored. In the present study, we sought to characterize the effects of tDMARD treatment on joints of early RA patients. Herein, we demonstrate that (a) tDMARD treatment improves disease by modulating the expression of a small number of genes, without affecting the expression of a vast number of other genes that are differentially expressed in early RA versus normal synovium, (b) gene co-expression modules correlate with tDMARD treatment and identify the major cell types that are acted upon by tDMARD components, (c) synovial biopsies from early RA patients can be classified into three major types, but these types are not associated with known clinical disease measures or response to tDMARD, and (d) T cell activation and plasmablast/plasma cell differentiation and function are the major pathways impacted by tDMARD. Furthermore, our results identify several pathways/targets (e.g., TWEAK and TLR7 as described below) that could be investigated further as alternative treatments or for use in combination with current therapies to further reduce disease activity and prevent irreversible damage.

Although several gene expression profiling studies have compared the global transcriptional differences between RA and OA synovial tissue biopsies from affected joints [16–18], genome-wide transcriptional differences between normal healthy synovium and early RA have not been explored. Many genes (>5000) were differentially expressed in early RA when compared to normal synovium with 2398 genes showing higher and 2961 lower expression in RA synovial tissue (Fig 1B). Top predicted pathways based on genes that were differentially expressed in RA compared to normal synovium were immune system, adaptive immunity, cell cycle, and immunoregulatory interactions between a lymphoid and a non-lymphoid cell (Fig 1C). Similarly, previous gene expression profiling studies have shown that adaptive immunity and cell cycle regulation genes are differentially expressed between RA and OA synovium [19]. The top upstream regulators of RA differentially expressed genes included IFNG, IL1B, TNF, CSF2, TNFSF12 and TLR7 (Fig 1D). Anti-TNF (multiple agents approved) and IL1B-inhibitors (anakinra) are approved for the treatment of RA. In addition, CSF-2 (GM-CSF) expression is increased in RA synovial tissue and elevated levels of GM-CSF protein are observed in RA synovial fluid and tissue samples [20, 21]. Furthermore, GM-CSF plays a major role in macrophage differentiation and activation and it has also been implicated in the differentiation and pathogenesis of Th17 cells [22–24]. Mavrilimumab, a monoclonal antibody that blocks GM-CSF signaling by targeting GM-CSFR alpha chain, has shown efficacy in phase two clinical trials in RA [25]. Because unbiased pathway analysis returned three proven targets of RA, targeting other significant upstream regulators, namely TNFSF12 (TWEAK) and TLR7 may also exhibit therapeutic efficacy. In agreement, studies providing some *in vitro* and *in vivo* validation for targeting TWEAK and TLR7, either as stand-alone or combination therapeutics have been published. The level of TWEAK is increased in RA synovium, serum, and synovial fluid. TWEAK induces osteoclastogenesis *in vitro* and *in vivo* [26], indicating that anti-TWEAK-based therapeutics may inhibit bone resorption and degradation observed in RA and more importantly, may also augment the efficacy of other RA therapeutics with non-overlapping modes of action. Given the observations that TLR7 knock-out mice displayed attenuated disease in murine collagen-induced arthritis (CIA) model [27] and intra-articular knockdown of TLR7 by an adenoviral antisense resulted in decreased disease activity in the rat CIA model [28], our observation that TLR7 is an upstream regulator of RA differentially expressed genes, further validates TLR7 as a potential target for RA. Therefore, a small molecule TLR7 antagonist may show therapeutic activity in RA.

The most unexpected finding of the study was that a very small fraction of early RA increased genes (146 out of total 2398 genes) were downregulated after 6 months of treatment with tDMARD, indicating that less than ten percent of the upregulated genes at baseline may account for moderate to good efficacy seen in patients in this early RA cohort (Fig 2A and 2B). The potential implications of this finding are several. On one hand, many differences remain between treated joints and healthy joints, which suggests that additional pathways could be targeted for greater efficacy. An alternative interpretation is that irreversible damage to synovium remains after disease onset and this damage may be resistant to any treatment. Finally, we could also posit that efficacious tDMARD treatment results in restricted changes in a small number of pathways or processes that result in disease improvement. Interestingly, we identified two gene co-expression modules (modules 10 and 17) that showed good correlation with tDMARD treatment (Fig 4A). Modules 10 and 17 contained genes involved in lymphocyte pathways, adaptive immune system, TCR signaling, and leukocyte adhesion and aggregation. Enriched pathways and processes for these modules contained genes required for T cell activation and signaling (e.g., *ZAP70*, *LCK*, *IL21R*, *CD8B*, *CD3D*, *CD3G*, *CD40LG*, *ICOS)* as well as plasmablast/plasma cell differentiation (e.g., *PRDM1*, *XBP1*, *IRF4*). These pathways are known to be important for RA disease pathogenesis as discussed below.

Although a number of immune cell types are present in the RA inflamed synovium, a majority of the synovial tissue infiltrating immune cells are T cells, particularly CD4+ T cells [29–31]. The identification of the shared epitope in RA susceptibility *HLA-DRB1* alleles that binds various arthritogenic peptides leading to T cell receptor-mediated breakdown of immunological tolerance to citrullinated proteins provides the most robust evidence for the involvement of CD4+ T cells in the pathogenesis of RA [32]. The identification of T cell activation and function genes (e.g., *PTPN22*, *PTPN2*, *CD28*, *CD2*, *CTLA4*, *STAT4*, *PRKCQ*, *IL2RA*, *IL2RB*) as risk alleles in genome-wide association studies [33] and the clinical efficacy of abatacept, a T cell co-stimulatory molecule/signal 2-targeted therapeutic [34] further strengthen the notion that RA is a T cell-mediated autoimmune disease. Given that tDMARD therapy down-regulated the expression of T cell activation and function genes in early RA (Figs 4A and 5C), it appears to be one of the underlying mechanisms of tDMARD therapeutic action.

Previous work has demonstrated that ACPA-specific plasmablasts were readily detectable in RA patients, even without *in vitro* stimulation, demonstrating the importance of plasmablasts in ongoing disease [35]. In addition, long-lived autoreactive plasma cells produce aberrant autoantibodies, which play a key role in the pathogenesis of autoimmune diseases such as RA and systemic lupus erythematosus (SLE) [36]. Therefore, targeted depletion of long-lived plasma cells has been proposed and is being tested in SLE patients [37]. Although there are no well-documented differentiation pathways related to short-lived plasmablast and long-lived plasma cells in databases such as IPA or KEGG, our manual analysis of plasmablast/plasma cell genes (*CD19*, *CD38*, *CD138*, *CD27*, *SLAMF7*, *PRDM1*, *XBP1*, *IRF4*, *IGJ*, *TNFRSF17*) offers a preliminary understanding of the differentiation-related genes in our samples. The down-modulation of plasmablast/plasma cell pathway by tDMARD treatment illustrates a novel potential mechanism of tDMARD beyond an anti-inflammation mechanism of action (Fig 4C). This may result from the lack of T cell help (given the down-modulation of T cell activation pathway) for ongoing B cell activation and differentiation into short-lived plasmablasts. Alternatively, tDMARD treatment may directly inhibit B cell differentiation via an unknown mechanism. More studies are warranted to further elucidate this mechanism *in vitro* or in preclinical studies. A larger number of subjects in future studies will also allow for a better understanding of the association between the plasmablast/plasma cell differentiation pathway activity and treatment response.

## Supporting information

S1 Table Composition of co-expression modules. Gene symbols are provided for the 20 derived co-expression modules.
(XLSX)

S2 Table Enrichment results from Reactome. Gene expression enrichment results are provided for the comparison of early RA baseline samples to normal controls and LDA RA samples after 6 months of tDMARD treatment compared to normal controls.
(XLSX)

S3 Table IPA predicted upstream regulators. Predicted upstream regulators from IPA are given for the comparison of early RA baseline samples to normal controls and LDA RA samples after 6 months of tDMARD treatment compared to normal controls.
(XLSX)
